# Quantifying and Interpreting Connection Strength in Macro- and Microscopic Systems: Lessons from Bell’s Approach

**DOI:** 10.3390/e24030364

**Published:** 2022-03-03

**Authors:** Christoph Gallus, Pawel Blasiak, Emmanuel M. Pothos

**Affiliations:** 1 THM Business School, Technische Hochschule Mittelhessen, D-35390 Gießen, Germany; 2 Institute of Nuclear Physics, Polish Academy of Sciences, PL-31342 Kraków, Poland; pawel.blasiak@ifj.edu.pl; 3 Psychology Department, University of London, London EC1V 0HB, UK; emmanuel.pothos.1@city.ac.uk

**Keywords:** Bell statistic, CHSH inequality, free choice, locality, propagation of information, causality, machine learning

## Abstract

Bell inequalities were created with the goal of improving the understanding of foundational questions in quantum mechanics. To this end, they are typically applied to measurement results generated from entangled systems of particles. They can, however, also be used as a statistical tool for macroscopic systems, where they can describe the connection strength between two components of a system under a causal model. We show that, in principle, data from macroscopic observations analyzed with Bell’ s approach can invalidate certain causal models. To illustrate this use, we describe a macroscopic game setting, without a quantum mechanical measurement process, and analyze it using the framework of Bell experiments. In the macroscopic game, violations of the inequalities can be created by cheating with classically defined strategies. In the physical context, the meaning of violations is less clear and is still vigorously debated. We discuss two measures for optimal strategies to generate a given statistic that violates the inequalities. We show their mathematical equivalence and how they can be computed from CHSH-quantities alone, if non-signaling applies. As a macroscopic example from the financial world, we show how the unfair use of insider knowledge could be picked up using Bell statistics. Finally, in the discussion of realist interpretations of quantum mechanical Bell experiments, cheating strategies are often expressed through the ideas of free choice and locality. In this regard, violations of free choice and locality can be interpreted as two sides of the same coin, which underscores the view that the meaning these terms are given in Bell’s approach should not be confused with their everyday use. In general, we conclude that Bell’s approach also carries lessons for understanding macroscopic systems of which the connectedness conforms to different causal structures.

## 1. Introduction

John Bell’s seminal work [1,2,3,4,5,6] has triggered a rich tradition of experimental studies [7,8,9,10,11,12,13] as well as theoretical and interpretational work [14,15,16,17,18,19,20,21,22,23,24,25,26,27,28,29,30]. To derive his famous inequalities, he has taken a realist worldview, making the additional assumption of free choice and locality, as formally defined by precise equations in a hidden variable model. The debate about the meaning of these assumptions and the experimental findings is still ongoing, with great intensity. This is partly due to the fact that the terms realism, free will and locality are cherished notions about which people hold passionate beliefs, based on their everyday experiences in the macroscopic world or assumptions about nature.

Assuming realism, the mathematical equivalence between violations of the free choice assumption and violations of the locality assumption was demonstrated in [27]. The present paper applies the approach in [27] to the setting of a macroscopic game. This has the advantage that it is fully describable in the language of everyday experiences, without the need to, e.g., specify what constitutes a measurement. The description of the game rounds is operational using terms that are neutral with respect to a particular philosophical worldview. On this basis, the mathematical equivalence of the resource consumption by optimal strategies that seek to generate a given statistical distribution can be shown.

The game, as presented here, uses four parties–Alice and Bob, a Quiz-Master posing questions and a separate Verifier, who has access to the complete statistics of the questions and answers. The description of the game allows two possible causes for violations of Bell inequalities at the level of the Verifier, namely, bribes and hidden communication devices. We use the neutral term *connection strength* to describe the close connection between the decisions by Alice and Bob (or alternatively the measurement results for entangled particles) as it manifests itself in the joint statistic in the hands of the Verifier.

Quantum entanglement provides a resource to achieve statistical distributions, which classically would require the use of cheats. Bell’s work and the CHSH quantities can be seen as providing statistical tools to investigate general causal models equally on the micro- and macroscopic level. We will use this point of view in two ways. On the one hand, we will discuss the possible use of Bell inequalities and CHSH quantities in macroscopic real life situations, outside the artificial game set-up. Here we will look at a situation from the financial world, where the dissemination and use of private information is regulated by laws to prevent insider trading and front running. In such cases, information barriers play a role and the behavior of market participants can be analyzed statistically using Bell’s approach. Interestingly, we will discuss how particular data patterns may be used to infer specific possible regulatory breaches. We will also briefly consider the role of causal mechanisms in social and financial set-ups, where contexts change over time, which makes the straightforward application of machine learning tools difficult. On the other hand, we will use the comparison with the macroscopic game to discuss interpretations of violations of Bell inequalities in quantum mechanical experiments, with a view to explicate concepts such as free choice, locality, contextuality and predictive completeness.

The present paper is organized as follows. In Section 2, we describe the macroscopic game in detail, including methods for the determination of the CHSH values and the situation where only a finite number of rounds can be played. In Section 3, we give examples of strategies where cheats are used selectively in some of the rounds. On this basis, two measures are defined based on optimal strategies, using cheats selectively to generate a statistic that wins the game. The equivalence of the measures is subsequently shown and a formula to compute their value from the CHSH-values alone is provided in a situation where non-signaling applies. Section 4 points to the use of entangled particles as an alternative resource offered by nature, which allows one to win the game without using the macroscopic cheats of communication or bribes. In Section 5, the possible use of CHSH values for discovering insider trading and front running in a financial set-up are discussed, as a macroscopic application of Bell’s approach. We conclude by discussing different terminologies and interpretations of connection strength with Bell’s approach in macro- and microscopic situations.

## 2. A Macroscopic Game

Bell statistics and the violation of CHSH inequalities can be explained in a game with two players, called Alice and Bob, as well as a Quiz-Master and a Verifier. Such presentations of Bell tests are often referred to as a CHSH game and have been given for different situations, such as hypothetical TV quizzes, polls or trials [24,31,32,33,34], in which the Verifier and the Quiz-Master are often seen as one person. In addition to allowing a description in everyday language using familiar macroscopic situations, games settings also have interesting applications, because CHSH games are rigid, so that strategies with maximum success probability are isomorphic [35,36,37].

We present the story from the view point of Alice and Bob, who have been invited to participate in a game over many rounds, with the possibility of winning a sizeable prize at the end. The game works as follows: Alice and Bob are locked up in separate rooms without any means of communication. They are confined therein for a long time to play numerous rounds of the game. In each round, the Quiz-Master poses a question *x* to Alice and a question *y* to Bob. These questions are generally taken from a list L={0,1,2,…,L} of possible questions. The Quiz-Master may, for example, pose a question by slipping a piece of paper with the question written on it under the door. Alice gives her answer *a* by pressing one of two buttons that are in front of her. So, we have a∈{±1} in each round and the answers are electronically sent to the Verifier. Similarly, Bob gives his answer b∈{±1}, which is also electronically transmitted to the Verifier. Alice and Bob have to give an answer in each round within a given time span, otherwise they lose automatically. During the game, Alice should not be informed about y,b and Bob should not be informed about x,a. Note that Alice and Bob may give different answers, if they receive the same question in different rounds.

Alice and Bob work as a team to try to win the game, by creating a high absolute number for a statistical quantity, called *S*, that is generated from their answers and the questions of the Quiz-Master. The precise definition of *S* is given below. Alice and Bob may meet before the commencement of the game and discuss a response strategy, for example, they may agree that *“in round n=15 Alice will answer a=1 if the Quiz-Master has written question x=0 with blue ink on the paper”*. They may also use randomized strategies or other means to decide which button to press. However, Alice and Bob are not allowed to communicate during the game.

**Figure 1:**
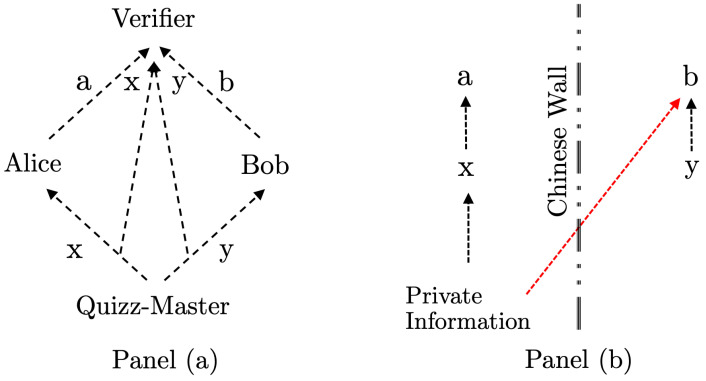
(**a**) The flow of information in the macroscopic game is shown by dotted lines in Panel (**a**). The flow of information does not necessarily represent causal influences as Alice and Bob may generate their answers a,b by using strategies that ignore information that they receive. (**b**) The right-hand panel shows the intended separation between private (insider) information (which should only be available for *a*) by a Chinese Wall in the financial example described in Section 5. Public information is not shown as it is available to all participants. The red arrow shows a breach of the Chinese Wall.

In this set-up, we distinguish between the Quiz-Master and the Verifier. The latter may be thought of as an objective and unbribable individual, like a public notary. The Verifier sees all questions and answers, whereas the Quiz-Master can freely choose x,y in each round, but does not see the answers a,b. The Verifier announces, after a finite number of rounds, whether Alice and Bob have won, possibly awarding attractive prizes. The number of rounds *N* is fixed and known to Alice and Bob before the start of the game. The flow of information is illustrated in Panel (a) of Figure 1.

After all electronic communication has been received (possibly with some significant delay), the Verifier has a list of quadruplets (a,b,x,y) from which he computes the statistic P(ab|xy) for the product value ab, conditional on different pairs of questions xy (in physical applications xy would be referred to as measurement settings). Here, we use the conventional notation of writing xy as shorthand for the question pair (x,y) and ab∈{±1} for the product value of *a* and *b*. Given these definitions, we can compute expectation values for the product ab, corresponding to different question pairs, xy:

$${\langle ab\rangle }_{xy}=\sum_{ab}\;ab\;P\left(ab\;|\;xy\right)$$

We will focus on the well-known case with just two possible questions L={0,1}. Under this assumption, there are four possible cases xy∈{00,01,10,11}. The list of answers (a,b) is thus partitioned into four sets (*regimes*) and in each regime an expectation value ${\langle ab\rangle }_{xy}$ can be computed for the product ab. We will assume that each question regime occurs at least once in the game, so that all four expectation values are well defined. Following the CHSH approach [38], the combination of these four expectation values yield four *S*-values

(1) $$\begin{array}{ccc} S_{1} & = & \;\;\;{\langle ab\rangle }_{00}+{\langle ab\rangle }_{01}+{\langle ab\rangle }_{10}-{\langle ab\rangle }_{11}\;, \end{array}$$

(2) $$\begin{array}{ccc} S_{2} & = & \;\;\;{\langle ab\rangle }_{00}+{\langle ab\rangle }_{01}-{\langle ab\rangle }_{10}+{\langle ab\rangle }_{11}\;, \end{array}$$

(3) $$\begin{array}{ccc} S_{3} & = & \;\;\;{\langle ab\rangle }_{00}-{\langle ab\rangle }_{01}+{\langle ab\rangle }_{10}+{\langle ab\rangle }_{11}\;, \end{array}$$

(4) $$\begin{array}{ccc} S_{4} & = & -{\langle ab\rangle }_{00}+{\langle ab\rangle }_{01}+{\langle ab\rangle }_{10}+{\langle ab\rangle }_{11}\;. \end{array}$$

It is agreed that Alice and Bob win the game if $S_{i}$ exceeds 2 for one i=1,2,3,4, after all rounds have been played.

The four expectation values used in the expressions above are essentially correlations between the answers a,b measured in the four different regimes. What makes winning the game hard is the fact that in the expression for any $S_{i}$, one of the correlations is subtracted from the other three. It is this feature of the $S_{i}$-values which precludes success using a simple strategy of agreeing, prior to the game, to answer questions in a specific way.

We will use these $S_{i}$-values when quantifying connection strength. Technically, some information is discarded by moving from the full statistic P(a,b|xy) to the *S*-values, as the value of the answer pair (a,b) is compressed into the product ab. However, we can do this without the loss of generality in relation to the issues which are the focus of the present work.

Typically, it is assumed that the number of rounds played is arbitrarily large, so that the empirically observed *S*-values are equal to their mathematical expectation. Then, it can be shown that in the game outlined above, without quantum resources, it has to hold that

(5) $$\begin{array}{ccc} |S_{i}|⩽2 & & \;\mathrm{for}\;\;i=1,\ldots ,4\;. \end{array}$$

For a realist macroscopic game, we assume that the number of rounds *N* is finite. For simplicity we focus on $S_{1}$. In a game with a finite number of rounds it is possible that Alice and Bob produce an $S_{1}$-value above 2 by pure luck, even if their strategy consists of nothing more than generating all their answers a,b from random coin tosses. As shown in [39], by choosing answers at random, the probability of generating $S_{1}$-values above 2 is

(6) $$\begin{array}{c} \mathrm{Pr}\left\{S_{1}⩽2+\eta \right\}⩾1-8e^{-N{\left(\eta /16\right)}^{2}} \end{array}$$

for any η⩾0. The right-hand side of the inequality requires a larger number of rounds to give meaningful bounds, so Alice and Bob will need to spend some time playing. Equation (6) implies that in a game of, e.g., N= 10,000 rounds, with a Verifier demanding $S_{1}>2.5$ to declare Alice and Bob winners, choosing answers at random, will get them a winning chance of less than 0.05%. This follows from using η=0.5 and N= 10,000 in Equation (6), which yields $\mathrm{Pr}\{S_{1}⩽2.5\}⩾0.99954$.

## 3. Winning by Cheating

After the game is explained to them, Alice and Bob realize that their chances of winning are very slim. Without knowing what question the other partner gets during the game, it seems impossible to win. After all, to score a high $S_{1}$-value, Alice and Bob should strive to generate a positive value for ab in the regimes xy=00,01,10 but a negative value for ab in the regime xy=11. As briefly noted above, the minus sign in the fourth term makes it impossible to achieve, e.g., $S_{1}>2.5$ by simply offering perfectly correlated answers. A regime-dependent strategy seems to be needed. However, when Alice only has the information x=1, when sitting in her room, how should she know which question regime xy∈{10,11} is used in the current round?

The questions arrive at Alice and Bob’s rooms without forewarning and each player has no way of informing their partner what question they have received and what answer they want to give. The problem is that Alice and Bob have no apparent capacity to communicate during the game, because they are isolated in their respective rooms. What can they do? With a classical understanding of the world and not believing in telepathy, they need to cheat and create a way to connect and infer the question regime, if they are to have a way of winning. Two cheating strategies come to mind:

**Cheat** **1:** A *secret communication device*, such as a smart phone, is smuggled into their isolated rooms.

Receiving question x=1, Alice may, for example, send a clandestine text message to Bob telling him what question she received. With this cheating method, they can pre-agree on an answer strategy. For example, Alice may always answer a=+1 and send a text message in every round, in which she has received the question x=1. Bob will answer b=+1 except for those rounds in which Bob receives the message “x=1” from Alice. When he gets that message, and therefore knows the applicable question regime for the current round, Bob will press the button b=+1, if y=0, but b=−1, if y=1. We assume that Bob receives all messages sent by Alice, but never sends a message himself. With this specific cheating strategy, Alice and Bob create the behavior shown in Table 1 at the level of the Verifier. The value of the product ab is always constant in each of the question regimes, so Bob and the Verifier will always compute $S_{1}={\langle ab\rangle }_{00}+{\langle ab\rangle }_{01}+{\langle ab\rangle }_{10}-{\langle ab\rangle }_{11}=1+1+1-\left(-1\right)=4$.

**Cheat** **2:** Although the Verifier is assumed to be untouchable, the Quiz-Master may be open to *bribes*.

The Quiz-Master may, for example, be bribed to let Alice and Bob have the complete list of questions that will be asked over the entire game in advance. If Alice and Bob have that list in their rooms, they know which question regime applies in any given round. They can then easily achieve $S_{1}=4$ by ensuring ab=+1 in the rounds with regimes xy=00,01,10 and ab=−1 in regime xy=11. Alice and Bob can, for example, create the behavior shown in Table 2, at the level of the Verifier. Here the value of the product ab is always constant in each of the question regimes, so the players and the Verifier will always compute $S_{1}={\langle ab\rangle }_{00}+{\langle ab\rangle }_{01}+{\langle ab\rangle }_{10}-{\langle ab\rangle }_{11}=1+1+1-\left(-1\right)=4$.

Possibly, things are not that easy and the Quiz-Master has to use a random number generator to generate the questions during the game, so there is no list of questions that could be shared in advance. Alternatively, the Verifier may send questions to the Quiz-Master, which he has to ask during the game. In this case, the cheating trio has to operate more subtly and the Quiz-Master has to clandestinely inform the players during the game about the other question posed in the current round. To achieve this he may use different ink or pre-agreed bit combinations in an electronic message to let, e.g., Bob know what question he has just given to Alice.

All these versions of Cheat 2 create knowledge with Alice and Bob regarding the question regime xy.

### 3.1. Measure $\mu_{1}$

Let us assume that Cheat 1 is a possibility, but bribing the Quiz-Master is impossible. Therefore, Alice and Bob have no prior knowledge of the question regimes in any round and cannot influence them. Let us also assume that using a smartphone is possible, but this has a certain risk of discovery, so that Alice and Bob will try to use it as rarely as possible. *We define $\mu_{1}$ as the maximal percentage of rounds in which Alice and Bob do not need to use the communication device when trying to create a desired statistic P(a,b|xy) at the level of the Verifier.*

**Example** **1.** *To illustrate this approach, we give an explicit example. We use the pre-agreed answer strategy described for Cheat 1 above, but such that it only applies in certain rounds, e.g., when Alice receives question* x=1 *and feels safe to send a text.*

*We can divide the number of rounds* $\Lambda =\left\{1,2,\ldots ,N\right\}=T\cup \bar{T}$ *into two sets. For the round belonging to set T Alice will send a text, but in the rounds* $\bar{T}$ *no text is sent. Note that this partition is only fully known to Alice after all rounds have been played. It is never known to the Quiz-Master, who does not know about the secret communication device.*

*This strategy with restricted communication yields the distribution of values for the product* ab *shown in Table 3. It is an extension of Table 1, but now the value of the product* ab *is no longer constant in each question regime. For* xy=11 *the value* ab=1 *occurs when no text message is sent, but* ab=−1 *occurs in the case of a text message. This yields*

$$\begin{array}{ccc} S_{1} & = & {\langle ab\rangle }_{00}+{\langle ab\rangle }_{01}+{\langle ab\rangle }_{10}-{\langle ab\rangle }_{11}\\ & = & 1+1+1-\left(1\cdot P(ab=+1\;|\;11)+(-1)\cdot P(ab=-1\;|\;11)\right)\\ & = & 3-P\left(ab=+1\;|\;11T\right)\cdot P\left(T\;|\;11\right)-P\left(ab=+1\;|\;11\bar{T}\right)\cdot P\left(\bar{T}\;|\;11\right)\\ & & +\;P\left(ab=-1\;|\;11T\right)\cdot P\left(T\;|\;11\right)+P\left(ab=-1\;|\;11\bar{T}\right)\cdot P\left(\bar{T}\;|\;11\right)\\ & = & 3-P\left(\bar{T}\;|\;11\right)+P\left(T\;|\;11\right)=3-\left(1-P\left(T\;|\;11\right)\right)+P\left(T\;|\;11\right)\\ & = & 2(1+P(T\;|\;11)), \end{array}$$

*because* P(ab=+1|11T)=0 *and* P(ab=−1|11T)=1 *, as Bob will always change his answer to* −1 *, if he receives a text, whereas* $P(ab=+1\;|\;11\bar{T})=1$ *and* $P(ab=-1\;|\;11\bar{T})=0$ *, as Bob will stick with his answer* +1 *, if he does not receive a text.*

*Therefore, the amount by which* $S_{1}$ *exceeds the bound of 2 is merely dependent on how often Alice sent a text in the question regime* xy=11 *. Note that Alice can control how often she sends a text, i.e.,* P(T)=P(T|10)+P(T|11) *, but not the probability* P(T|11) *, because Alice does not know the question that Bob has received, when she has to decide whether to send a text or not.*

### 3.2. Measure $\mu_{2}$

Let us assume that Cheat 2 is a possibility, but using communication devices during the game is out of question. Let us also assume that using Cheat 2 has a certain risk of discovery for the Quiz-Master, so Cheat 2 should not be used in every round. *We define $\mu_{2}$ as the maximal percentage of rounds in which Alice and Bob do not need knowledge of the applicable question regime when trying to create a desired statistic P(a,b|xy) at the level of the Verifier.*

**Example** **2.** *To illustrate this approach we also give an explicit example. We use the pre-agreed answer strategy described for Cheat 2 above but assume that the Quiz-Master lets Alice and Bob know the question regime only in certain rounds. The standard answer for Alice and Bob is thus* +1 *, but if they know that in the current round* xy=11 *applies, Bob will change his answer to* −1 *, whereas Alice will not change hers.*

*Similarly to Example 1, we can divide the number of rounds* $\Lambda =\left\{1,2,\ldots ,N\right\}=K\cup \bar{K}$ *into two sets. For the round belonging to set K, both Alice and Bob will know the value of* xy *, but in the rounds* $\bar{K}$ *none of the partners have that knowledge. This division can be known in advance, if the Quiz-Master has a pre-generated list of questions that he is willing to share.*

*This strategy yields the distribution of values for the product* ab *shown in Table 4. Again, the value of the product* ab *is no longer constant in the regime* xy=11 *. The formal computation is isomorphic to the computation in Example 1:*

$$\begin{array}{ccc} S_{1} & = & {\langle ab\rangle }_{00}+{\langle ab\rangle }_{01}+{\langle ab\rangle }_{10}-{\langle ab\rangle }_{11}\\ & = & 1+1+1-\left(1\cdot P(ab=+1\;|\;11)+(-1)\cdot P(ab=-1\;|\;11)\right)\\ & = & 3-P\left(ab=+1\;|\;11K\right)\cdot P\left(K\;|\;11\right)-P\left(ab=+1\;|\;11\bar{K}\right)\cdot P\left(\bar{K}\;|\;11\right)\\ & & +\;P\left(ab=-1\;|\;11K\right)\cdot P\left(K\;|\;11\right)+P\left(ab=-1\;|\;11\bar{K}\right)\cdot P\left(\bar{K}\;|\;11\right)\\ & = & 3-P\left(\bar{K}\;|\;11\right)+P\left(K\;|\;11\right)=3-\left(1-P\left(K\right)\;|\;11\right))+P\left(K\;|\;11\right)\\ & = & 2(1+P(K\;|\;11)). \end{array}$$

*Therefore, the amount by which* $S_{1}$ *exceeds the bound of 2 is merely dependent on the number of rounds in which the question regime* xy=11 *is known. Note that if the Quiz-Master does not share information in all rounds, then the above strategy may be suboptimal. This is because with the above strategy, knowing the question regime is only valuable to the players in the case of* xy=11 *. If the Quiz-Master deliberately shares the regime* xy *mainly in rounds with* xy≠11 *, Alice and Bob have to think about a different answering strategy.*

### 3.3. Measuring Connection Strength

Both cheats create a connection between Alice’s and Bob’s side in certain rounds and the Examples 1 and 2 have a similar formal structure. Are the cheats equivalent?

At first glance, both Cheat 1 and 2 somehow imply that at least either Alice or Bob knows what question their partner has received in a certain round. However, the cheats are not equivalent. Cheat 1 is subject to the speed limit for classical communication, whereas Cheat 2 is not. Imagine Alice, Bob and the Quiz-Master living on three different space stations sitting on a straight line (the Quiz-Master’s space station being in the middle). Cheat 1 may now become useless if Alice and Bob only have a short time to press the answer button after receiving the question. Cheat 2, with a pre-disclosed list of questions or with the Quiz-Master secretly sharing the entire regime in the message that contains the question, is not dependent on the speed-of-light limit.

Furthermore, Cheat 1 means that, e.g., x,a may be revealed to Bob. By contrast, Cheat 2 allows Alice to know *y* and Bob to know *x*, whereas the answers a,b cannot be shared without a communication device between Alice and Bob, because the Quiz-Master does not have information about the given answers, as illustrated in Panel (a) of Figure 1. Under the Cheat 2 scenario, Alice and Bob may, of course, pre-agree upon the answers for each round when the regime is fully known to at least one of them, so if Alice and Bob trust each other to follow the strategy, the answers a,b can be inferred by both players. However, in the case that Alice or Bob gets confused and deviates from the strategy, the inferred answers will not be equal to the answers that were actually given to the Verifier, which is another way of saying that Cheat 2 is not equivalent to having a communication device in the rooms.

Despite the fact that the cheats are not equivalent, it is possible to prove the mathematical equivalence of the two measures $\mu_{1}$ and $\mu_{2}$. Assuming an arbitrarily large number of rounds *N*, so that the empirically observed *S*-values equal the mathematical expectation, the following theorem can be proved:

**Theorem** **1.** *(a) For any statistic* P(a,b|xy) *that shall be created at the level of the Verifier, the two measures are fully equivalent, that is,* $\mu_{1}=\mu_{2}$ *.*

*(b) For any statistic* P(a,b|xy) *satisfying the so-called non-signaling condition*

(7) $$\begin{array}{ccc} P\left(a|x0\right)\;=\sum_{b\in \{\pm 1\}}P\left(a,b|x0\right) & = & \sum_{b\in \{\pm 1\}}P\left(a,b|x1\right)\;=\;P\left(a|x1\right)\;\;\mathrm{for}\;\mathrm{all}\;a,x, \end{array}$$

(8) $$\begin{array}{ccc} P\left(b|0y\right)\;=\sum_{a\in \{\pm 1\}}P\left(a,b|0y\right) & = & \sum_{a\in \{\pm 1\}}P\left(a,b|1y\right)\;=P\left(b|1y\right)\;\;\mathrm{for}\;\mathrm{all}\;b,y, \end{array}$$

*that shall be created at the level of the Verifier in the case of a game with two questions and two answers, we have that*

$$\mu_{1}\;=\;\mu_{2}\;=\;\left\{\begin{array}{ccc} \frac{1}{2}\left(4\;-\;\max\{\right|S_{1}\left|,\right|S_{2}\left|,\right|S_{3}\left|,\right|S_{4}|\})\;,\; & & \mathrm{if}\;S_{i}>2\;\;\mathrm{for}\;\mathrm{one}\;i\;,\;\\ 1\;, & & \mathrm{otherwise}\;. \end{array}\right.$$

**Proof.**  We present a short sketch of the proof (details are given in [27]), where the presentation is made in terms of physical Bell experiments, using the notions of Bell locality and free choice. The formal argument works equally well for the macroscopic game presented here. It is based on the decomposition of the desired statistic into

(9) $$\begin{array}{c} P\left(a,b|xy\right)\;=\;\sum_{\lambda \in \Lambda }P\left(a,b|xy\lambda \right)\cdot P\left(\lambda |xy\right)\;, \end{array}$$

assuming some a priori unknown hidden variable set Λ. For a given statistic, writing Equation (9) is always formally possible; see [24,40,41].

Not every formal way of writing Equation (9) can be translated into a strategy that is executable by Alice and Bob, but the generic Equation (9) includes all conceivable strategies that Alice and Bob may have agreed upon, before the commencement of the game. The expression P(a,b|xyλ) means that the probability of a certain value ab results not only from the question regime but also from an unknown cause λ, which can be one of the cheats described above or something else.

The model implicitly describes the distribution of questions chosen by the Quiz-Master for the different rounds through the standard formula

(10) $$\begin{array}{c} P\left(xy\right)\;=\;\sum_{\lambda \in \Lambda }P\left(xy|\lambda \right)\cdot P\left(\lambda \right)\;. \end{array}$$

The set Λ can be partitioned into two sets depending on whether

(11) $$\begin{array}{c} P(a,b|xy\lambda )\;=P(a|x\lambda )\cdot P(b|y\lambda )\; \end{array}$$

holds or not. Defining

$$\Lambda_{1}=\left\{\lambda \in \Lambda \;|\;\mathrm{Equation}\;(11)\;\mathrm{holds}\;\mathrm{for}\;\mathrm{all}\;x,y\right\}$$

we obtain a partition $\Lambda =\Lambda_{1}\cup \left(\Lambda \backslash \Lambda_{1}\right)$. Here, $\Lambda_{1}$ corresponds to the rounds where no communication and no bribes are used, and $\Lambda \backslash \Lambda_{1}$ corresponds to rounds where Cheat 1 was applied. A different partition is built on the question whether

(12) $$\begin{array}{c} P(xy|\lambda )\;=P(xy)\;\;\;\;\;\;\;(\mathrm{or}\;\mathrm{equivalently}\;P(\lambda |xy)\;=P(\lambda )), \end{array}$$

holds or not. Defining

$$\Lambda_{2}=\left\{\lambda \in \Lambda \;|\;\mathrm{Equation}\;(12)\;\mathrm{holds}\;\mathrm{for}\;\mathrm{all}\;x,y\right\}$$

we get a different partition $\Lambda =\Lambda_{2}\cup \left(\Lambda \backslash \Lambda_{2}\right)$. Here, $\Lambda_{2}$ corresponds to the rounds where no communication and no bribes are used, and $\Lambda \backslash \Lambda_{2}$ corresponds to rounds where Cheat 2 was applied.

To prove (a) we can employ a bijective construction, writing the desired statistic P(a,b|xy) as a convex combination of a statistic that does not use cheats and a statistic where either Cheat 1 or Cheat 2 is used. Formally, this is expressed by λ being in different parts of the partition, with the condition that the desired statistic is retained as the marginal distribution. This gives

$$\begin{array}{ccc} P(a,b|xy) & = & p_{1}\cdot P_{1}\left(a,b|xy\right)+\left(1-p_{1}\right)\cdot {\tilde{P}}_{1}\left(a,b|xy\right)\\ & = & p_{2}\cdot P_{2}\left(a,b|xy\right)+\left(1-p_{2}\right)\cdot {\tilde{P}}_{2}\left(a,b|xy\right), \end{array}$$

where $P_{1}$ is a statistic that can be created without using any cheats and ${\tilde{P}}_{1}$ is a statistic that can be created by Cheat 1 alone without bribing the Quiz-Master, whereas $P_{2}$ is a statistic that can be created without using any cheats and ${\tilde{P}}_{2}$ is a statistic that can be created by Cheat 2 alone, without the need of a communication device. The numbers $p_{1},p_{2}\in \left[0;1\right]$ have to be chosen suitably; see the Proof of Theorem 1 in [27].

To show (b), the desired statistic is written as a convex combination between the statistics of a PR-box and a statistic that can be created without using any cheats; see [42], Section 9.4 in [24] and the proof of Lemma 3 in [27]. □

Part (a) of Theorem 1 is a formal, model-independent equivalence concerning optimal strategies to generate a given distribution at the level of the Verifier. It shows that the measure $\mu_{1}$, i.e., the number of rounds in which the players can work without communication assuming an unbribable Quiz-Master, gives the same result as the measure $\mu_{2}$, i.e., the number of rounds in which the Quiz-Master can keep the question regime xy hidden from Alice and Bob, assuming that communication between Alice and Bob is impossible.

Part (b) of Theorem 1 allows the explicit computation of the values for the two measures from the *S*-values alone, assuming an additional condition. This additional condition stated in Equations (7) and (8) in part (b) of Theorem 1 is often referred to as the *non-signaling* condition. In particular, part (b) of Theorem 1 implies that in order to create a PR-box, that is a non-signaling statistic for which one of the CHSH expressions in Equations (1)–(4) reaches the maximal algebraic value of $|S_{i}|=4$, see [42], the players have to cheat in every round. However, note that in the examples of Table 3 and Table 4 non-signaling is violated, because 0=P(b=−1|01)≠P(b=−1|11). Alice and Bob may, of course, resort to randomization and the use of more complex strategies in their use of Cheat 1 or Cheat 2 to generate a statistic that looks less suspicious to the Verifier.

## 4. Winning with Additional Resources from Nature: Entangled Particles

Let us again assume L=1 for simplicity, so there are just two possible questions. We assume that Alice and Bob are allowed to take a measurement device and a number of systems of pairwise entangled particles, each described by a two-qubit Bell state

(13) $$\begin{array}{c} |\phi \rangle =\frac{1}{\sqrt{2}}\left({|0\rangle }_{A}{|0\rangle }_{B}+{|1\rangle }_{A}{|1\rangle }_{B}\right), \end{array}$$

into their room. The first component shall be in the room with Alice and the second in the room with Bob. Here, 0 and 1 are the two basis states used; see [24,43]. The entangled particles give a resource to Alice and Bob that allows them to win the game without using Cheat 1 or 2; see, for example, [33].

The availability of entangled particles does not mean that Alice and Bob have a communication device, because under the non-signaling condition a shared quantum state does not provide a means of communication. The availability of entangled particles is therefore not a use of Cheat 1, but a separate resource that needs to be understood differently. Of course, there are interesting classical strategies if some communication, consisting of one classical bit per round between Alice and Bob, is allowed, to simulate the statistics of the maximally entangled state in Equation (13), see [44]. So far, the challenge issued to Bell deniers in [24,39] to write a program for two computers, which would take the roles of Alice and Bob and which can win the CHSH game by producing significant violations of Bell Inequalities without cheating, has not been met by anyone.

As the collaboration of the Quiz-Master is not required for the use of entangled particles, winning with qubits is not a case of Cheat 2 either. However, with the additional resources at their disposal (assuming that they have an entangled pair of particles for each round of the game), Alice and Bob may pre-agree on a winning strategy, pushing the $S_{1}$-value at the level of the Verifier up to $S_{1}=2\sqrt{2}\approx 2.828$. Under this set-up, Alice and Bob become operators who configure their measurement device according to the question which they have received and who subsequently read out the result of the measurement and then press the answer button according to the measurement result shown by the apparatus.

Although the existence of such a resource has been shown experimentally [8,9,10,11,12], the open question is, of course, how to explain the inner workings (if any) of this resource from a realist worldview. This will be discussed briefly in Section 6.

## 5. Social and Financial Systems

Bell’s approach can also be useful to analyze connection strength in the macroscopic world. To do this, we move away from the somewhat artificial game presented in Section 2 and give an example from the financial world. The following example could be re-phrased for different social and strategic situations; cf. [45].

In the financial world, information and its dissemination play a key role. Information that is not known to other market participants may give a significant and possibly unfair advantage to some market participants. Knowledge about the financial data of a company, prior to the official release of such data to the public, would, for example, allow buying and selling with advantageous risk-return characteristics. Knowledge about the execution of a large order would allow traders to position themselves accordingly in advance, usually to the detriment of the economic beneficiary, who is behind the large order. To foster trust in the capital market mechanism and to ensure a fair playing field, many countries have adopted laws and regulations against insider trading or front running to prevent such practices.

These unfair practices can, for the purpose of this work, be characterized as a situation where an outside trader acquires *private information* about company data or a large order and acts upon it, by performing some specific market activity. To prevent such activities, asset managers and banks use so-called *Chinese walls* to separate people with legitimate access to private information from those that should not obtain private information. It is important to ensure that information does not leak across those Chinese walls. We would like to illustrate how information leaks that are exploited leave traces in an observed statistic, using the *S*-value by means of an analogy with the macroscopic game set-up presented in Section 2.

When using private information in an illegitimate way, positions of extraordinary sizes in unusual securities or atypically short holding times often arise. What is considered extraordinary and what is considered typical depends on the context, as well as the roles and normal activities of a market participant. In the setting of this paper, we assume that such a distinction can be made. We denote by +1 the execution of a typical activity by a market participant and by −1 the execution of atypical transactions on a given day. In an analogy to the macroscopic game, the market activities of Alice and Bob are recorded by a,b∈{±1} for each round of the game. A round may be an entire trading day or a shorter timeframe.

Market participants have certain information at their disposal, which may consist of publicly known facts, rumors and instructions from their managers. We summarize this information in just two categories, 0 and 1, and identify it with the questions x,y that Alice and Bob are asked in the macroscopic game. The activities a,b can, in a way, be seen as answers to the available information x,y.

We assume that Alice works for a large investment firm and is active in the market on a daily basis, executing only typical and permitted transactions, i.e., we have a=+1 on any day. The information that Alice receives *x* includes, on some days, instructions from her boss to execute large orders. On the days with x=0 she receives only public information, but on days with x=1 she also receives a large order from her boss, which constitutes private information. Because executing orders, including large ones, is a normal course of business for Alice, her activity would be classified as a=+1 even on days with x=1.

Bob does not work for the investment firm, and should not receive any private information. His daily information *y* consists of well-known economic facts, as well as rumors. On days with y=0 he receives only public information about general economic facts, but on days with y=1 there are also market rumors about large orders. Those rumors may be true or not; Bob does not know. We assume that Bob does not trade on the basis of rumors. However, when he hears a rumor he may sometimes call a friend on the private side at Alice’s firm, asking her whether the rumor is true. We assume that his friend does not lie to Bob and that she sometimes gives him confirmation that a rumor is true. Such confirmation would constitute a transfer of private knowledge (“*K*”). When this happens Bob will (illegally) front-run the order and execute unusual trades in the market, i.e., create the answer b=−1; see Panel (b) in Figure 1 for an illustration.

Can this behavior be detected? The situation described here yields the statistic shown in Table 5, which is isomorphic to Table 4. With a conventional view, detecting Bob’s illicit behavior is not easy. Private conversations by Bob, where private knowledge has been transferred, are generally difficult to prove. If we statistically look only at the days on which market rumors existed, i.e., at days with y=1, then Bob’s behavior consists of b=+1 as well as b=−1, so the unusual trades are washed out in the average ${\langle b\rangle }_{y=1}$. If we align this with data from Alice’s firm and look only at the days where a larger order existed, we obtain the regimes xy∈{10,11}; we can compute the averages ${\langle b\rangle }_{10}$ as well as ${\langle b\rangle }_{11}$, but this may not tell us much either.

Clearly, the above statistic violates non-signaling, as given in Equation (8), because Table 5 implies 0=P(b=−1|01)≠P(b=−1|11). Computing the $S_{1}$-quantity, we obtain the same result as in Example 2:

$$\begin{array}{ccc} S_{1} & = & {\langle ab\rangle }_{00}+{\langle ab\rangle }_{01}+{\langle ab\rangle }_{10}-{\langle ab\rangle }_{11}\\ & = & 2(1+P(K\;|\;11)). \end{array}$$

So the amount by which the $S_{1}$-value exceeds 2 is a function of the probability P(K|11), i.e., it depends on how often there was a transfer of private knowledge (*K*) to Bob on days where Alice had a large order and where rumors about such a large order existed in the market.

$S_{1}$-values above 2 help us identify changes in Bob’s behavior. If no such changes happen, i.e., if Bob never acts upon private information, $S_{1}=2$ would be the result. This is useful because legal problems typically arise when market participants act upon private information. It is interesting that Bell’s approach and the observed $S_{1}$-values are sensitive to P(K|11), even when there is no provable record of a direct inappropriate information transfer. Having applied such a model, observing $S_{1}>2$ can be seen as a warning sign.

Creating the data table with a quadruplet (x,y,a,b) for each day would, in principle, be possible for a verifying auditor, as she can find out whether x=1 holds from insider lists and documentation around orders at Alice’s firm. Regarding Bob, the determination of the value *b* for a given day would also be possible on the basis of potentially suspicious trading patterns that deviate from Bob’s normal activities. The value of *y* depends on whether rumors existed in the market on a given day. This can, in principle, be established without Bob’s collaboration, by analyzing recorded news feeds and questioning other market participants.

In practice, the Verifier could first check if the non-signaling condition of Equations (7) and (8) is violated before looking at the *S*-values. However, even if non-signaling is violated, that does not necessarily mean that a profitable violation of regulations around insider trading and front running has taken place. It may well be that Bob is merely acting on rumors and using his gut feeling regarding the validity of a rumor. To illustrate this case, we may, for example, assume that in the case of an unfounded rumor Bob makes a transaction with a probability of *p* but in the case of a true rumor he makes a transaction with a probability of $p^{\prime }$. This produces the behavior shown in Table 6.

In case of $p\neq p^{\prime }$, Bob behaves differently in the regime xy=01 than in the regime xy=11, so the non-signaling condition (8) is violated. Based on Table 6 we compute

$$\begin{array}{ccc} S_{1} & = & {\langle ab\rangle }_{00}+{\langle ab\rangle }_{01}+{\langle ab\rangle }_{10}-{\langle ab\rangle }_{11}\\ & = & 1+\left(-p+\left(1-p\right)\right)+1-\left(-p^{\prime }+\left(1-p\prime \right)\right)=2+2\left(p^{\prime }-p\right), \end{array}$$

so $S_{1}>2$ only occurs if Bob has the right gut feeling when acting on rumors, i.e., if $p^{\prime }>p$. Interestingly, the probability of whether rumors are objectively true more often than not, i.e., whether P(xy=11)>P(xy=01) holds, does not enter into this calculation.

So how can we tell whether Bob is simply an astute market participant, who has good judgement when hearing rumors, versus someone who is making unusual transactions based on insider information? The difference is that with Table 5, Bob never engages in unusual transactions when a rumor is unfounded, but with Table 6 this would happen with a probability of *p*.

## 6. Discussion: Connection Strength with Bell’s Approach

We considered Bell’s approach as a measure of connection strength in both micro- and macroscopic systems. We use the neutral term *connection strength* to describe the connection between the decisions of Alice and Bob, or alternatively the measurement results between two entangled particles, or, more generally, the results of measurements on two components of a micro- or macroscopic system, as shown in the joint statistic P(a,b|xy) of the Verifier. The goal of the approach is to put testable constraints on causal models that aim to explain or predict macro- or microscopic phenomena.

Under Bell’s approach, the Verifier starts with a full data table, i.e., a list in the form ${\left\{\left(n,a,b,x,y\right)\right\}}_{n=1,\ldots ,N}$. In creating this table, identifying rounds and dealing with rounds in which less than two answers were recorded can be problematic. In physical Bell experiments, e.g., with entangled photons, the time stamp is used to identify pairs of particles and thus assign questions and answers to each round of the game. With inefficiencies in photon detectors, it may happen that, e.g., a pair (x,a) for Alice has no counterpart on Bob’s side, which gives rise to the detection loophole [46,47]. Depending on the specific set-up, similar issues may arise in macroscopic systems as well. In macroscopic systems, either time stamps or ensembles of separated events happening at the same time can be used to generate the data table. It is desirable to ensure during the set-up process that no gaps occur, because if gaps occur due to a systematic cause, the subsequent analysis can be invalidated. In the macroscopic game described in Section 2, the Verifier can force Alice and Bob to give answers to all questions by simply ruling that Alice and Bob lose automatically if all answers are not received within a given timeframe.

In the next step, the Verifier reduces the full data table to the statistic P(a,b|xy), which is computed based on relative frequencies. Using the statistic P(a,b|xy), with properties as from Equations (7), (8), (11) or (12) can be tested. This is even advisable for quantum experiments, in which the non-signaling property is usually taken for granted [48,49,50]. According to Theorem 1, it is always the case that the measures $\mu_{1}$ and $\mu_{2}$ are equivalent, but computing their value on the basis of the *S*-values relies on the non-signaling condition, that is, Equations (7) and (8) have to hold. In macroscopic systems, the non-signaling condition cannot be taken for granted. In fact, the simple strategies presented in the examples shown in this paper violate non-signaling, but it would be possible for Alice and Bob to randomize those strategies so that the statistic appears less suspicious to the Verifier.

In a subsequent step, the Verifier moves away from the full statistic to the expectation values for the product ab under different regimes, i.e., to ${\langle ab\rangle }_{xy}$ with xy∈{00,01,10,11} and from there to the four *S*-values, $S_{1},S_{2},S_{3},S_{4}$. The important part is that the *S*-values provide the possibility of testing the validity of postulated, well-defined causal relationships. In Bell’s approach, the inequalities (5) are derived mathematically starting from three formally well-defined conditions, which are termed “*realism*”, “*locality*” and “*freedom of choice*”. The connection between causal interaction and Bell’s approach has been noted a couple of times [41,51]. The belief that every effect must have a cause has very strong roots in philosophy and science. A substantial part of the discussion and excitement around the interpretation of quantum theory comes from the expectation that locally-causal models for observed behavior have to be possible at the microscopic level as well.

In the macroscopic domain, humans have a desire for causal understanding and are not content with merely observing correlations. The morning cry of the rooster does not cause the sun to rise, as Judea Pearl put it, and causal relationships are important when it comes to understanding the consequences of human interventions [41], just as waking up the rooster early does not result in additional hours of daylight. Today, the causal structure behind the sunrise in the morning is known, so there is both a quantitative and qualitative causal understanding of this macroscopic physical phenomenon. With quantum theory, a mathematical description is known that allows the correct prediction of experimental quantities. Does there have to be a causal structure beyond such a mathematical description? In contrast to the precise description of macro-physical reality offered by classical mechanics and general relativity, social and economic models are not as successful in predicting behavior, but identifying reliable underlying causal relationships is equally important, for example in the area of interventions and policy-making.

In this paper we focus on *S*-values using Bell’s approach. There are alternative measures of connection strength, for example, those provided via the approaches of Legett, Garg, Suppes, Zanotti [14,15,17] and of Klyachko et al. [18]. Generally, instrumental inequalities provide bounds for given causal models, as shown by Pearl [41,52,53]. However, the *S*-values seem to provide a good approach to run tests on real data for assumed causal relationships. In a precise sense, the *S*-values and the corresponding CHSH inequalities give essentially the only interesting boundary hyperplanes in the local-realist non-signaling polytope in a situation in which we consider two parties, two possible questions and two possible answers [24,39,40]. As shown in Part (b) of Theorem 1, when non-signaling applies, knowing all four *S*-values even allows the computation of the minimum percentage of violations that is needed under optimal strategies. Although raw data can, of course, as a matter of principle, provide more information than data in a reduced form, essential points about causal relationships or the propagation of information may be hard to spot when looking at raw data, but can be seen directly using the *S*-values, as Bell’s approach teaches us.

### 6.1. Connection Strength in Macroscopic Systems; Elements of Reality

Connection strength may, in the macroscopic game, result from pre-agreement upon strategies and exploiting the available flow of information when implementing those strategies. In the game presented in Section 2, the answers a,b are generated by conscious decisions taken by Alice and Bob. In each round they have to press an answer button and may use the available information, as well as previously agreed-upon protocols, using a strategy such as “*to determine your answer a in round n = 120 in case you receive the question 1, toss a coin with probability of a=+1 equal to 44%*”, to decide which answer button to press. However, Alice and Bob may also decide to ignore information and previous agreements and choose on a whim. In the example of Section 5, Bob may suddenly feel nervous and refrain from executing a front-running strategy in the market, even when the market rumor was confirmed to him by an insider. In that sense, Alice and Bob are deemed to enjoy a freedom of choice that entangled particles in quantum mechanics, bound by conservation laws, do not possess. Nevertheless, a pre-agreed-upon strategy can be seen as a causal mechanism to explain and predict answers. However, to a realist, who believes in free choice, human decisions generally do not form an element of reality before they are made. Even if Alice and Bob have pre-agreed upon a strategy on how to press their buttons and even if we assume that Alice and Bob do not want to deviate from that strategy, so that their answers could be predicted in advance, would we say that their behavior is an element of reality in the sense of EPR [54], before the game was played?

With the deterministic theories of classical mechanics and general relativity the situation is different. Here, the time evolution of a macroscopic system is uniquely described from the initial conditions and the physical laws given by differential equations. This allows prediction with certainty and provides clear elements of reality. The experimentally observed connection strength results from these laws.

### 6.2. The Hidden Variable Space

A similar description seemed desirable to Einstein for the quantum level. If the quantum mechanical description is incomplete, it might be possible to complement it by finding additional variables and laws so that the statistical description offered by quantum theory is retained. In [3] Bell asks, “*can we not find another hidden-variable scheme with the desired local character?*”. Bell’s approach to a completion of quantum theory rests on an assumption called *realism*, which is typically identified with the mathematical assumption of a hidden variable space Λ, like the one shown in Equation (9). This space Λ is the starting point for discussing alternative models, as well as the concepts of locality and freedom of choice in the sense of Bell.

Although Λ can be formally defined as an explanatory model by the Verifier in an ex post analysis, Equation (9) does not mean that Λ is generally known in advance, as the macroscopic game shows. Winning the macroscopic game by means of classical strategies means that the mechanism assumed for a fair game had to be violated somewhere, but not everything can be derived from the statistic alone; therefore, supplementary knowledge may be required. For example, if the Verifier learns from other sources that the Quiz-Master has been bribed, it is clear how the violation could have ocurred. If the Verifier is certain that no communication device could have been used by Alice or Bob, because he ensured time-space separation, and if he sees $S_{1}=2.5$, can he conclude that the Quiz-Master has been bribed? Not necessarily, as a resource of entangled particles would be enough to create such a high *S*-value, although in Newtonian times one might have said that this can only be achieved through bribes, communication or something like witchcraft.

Formalizing the assumption of realism through the assumption of a hidden variable space Λ is a non-trivial step. It may require additional thought, at least with regards to the point in time of an experimental process at which such a Λ can be meaningfully introduced. The Newtonian concept of a universal time had to be given up, and a universal Λ may not necessarily be possible either. Without such qualifications, the term “realism“ is extended by making additional claims about universality, which not every realist may share.

In the proof of Theorem 1, the space Λ is partitioned into $\Lambda_{1}\cup \left(\Lambda \backslash \Lambda_{1}\right)$ as well as into $\Lambda_{2}\cup \left(\Lambda \backslash \Lambda_{2}\right)$. However, in the situation of the macroscopic game, this partition is only possible on an ex post basis with knowledge of the rounds in which a specific cheat has been used. This partition could possibly be carried out by Alice and Bob once all rounds have been played, but it would be beyond the ability of the Verifier to assign specific rounds to $\Lambda_{1}$ or $\Lambda_{2}$. Even to Alice and Bob, it would only be possible ex post facto, because, for example, Alice may decide on a whim in which round she feels safe to use her hidden mobile phone.

In economic and social systems, ensuring space-time separation to prevent Cheat 1 is usually unrealistic, so separation needs to rather come from information barriers such as Chinese Walls or from the use of unbroken encryption to prevent eavesdropping and the unintended dissemination of information. Nevertheless, Bell’s approach can be useful in these situations as well, as described in Section 5. Under the assumption of full compliance with Chinese Walls, certain statistical patterns should not occur. So an auditor, who comes in ex post facto as a Verifier, can in principle pick up signs of cheating based on the statistics alone. The space Λ can cover different models of conceivable cheating strategies.

As physicists are used to theories with impressive predictive power, there could be hope that for quantum mechanics Λ can be fixed objectively in advance by means of a hidden variable theory. With work on social or economic systems, one is used to lower expectations for precise predictions. There, retroactive explanations are sometimes offered, of which several can be designed ex post, because several Λ spaces can produce the same observed statistics. As an additional complication, a widely accepted predictive theory, i.e., a Λ publicized in advance, can be self-defeating, because people can choose to behave differently when they hear about the theory. In finance, known statistically significant traits in a market can disappear over time, when market participants try to exploit them.

### 6.3. Causality, Connection Strength and Explainability in Machine Learning

Finding reliable patterns to forecast the values of a given system is a task for which machine learning algorithms are increasingly applied; see, for example, [55,56,57,58,59,60,61]. This works well when the task is set in a static environment, such as optical character recognition or the classification of pictures with animals or human faces. It also works well when applied to games such as go or chess or when vehicles are learning to move autonomously. In these tasks the environment changes only very slowly over time and there are many examples of these training situations. Handwriting and printing styles and the characteristic appearance of animals or human faces do not change much over even reasonably long time periods. The rules of go and chess remain constant, and the physical laws and properties of a vehicle and its engines remain constant, when one is learning autonomous driving or flying, even if the actual surroundings are changing.

Social, economic, business and financial systems provide a greater challenge for machine learning as they involve complex conscious human decisions and changes in rules, often over a relatively short period. Usually for these complex systems, the training examples are few, compared to the numerous paths that the development of the system could take. In these cases, it can be helpful to complement the statistical learning process through the use of human contextual knowledge. For example, financial time series data for a company contain variables such as prices, earnings and sales, which may have a context-dependent interpretation by market participants. A surprising increase in sales and earnings is typically a good thing, but in regimes where market participants are concerned about overheating, a series of strong sales figures may not be so well received. Thus, the strength of the connection between variables will vary over time depending on the regime. In theory, given enough time, machine learning algorithms used for the automated processing of newsflows could learn to categorize different macro-regimes and react to news in a regime-dependent manner. However, in the recent past only a few economic cycles would be available for training and the distant past is generally different from today in too many aspects to be helpful for training. Therefore, for machine learning applications to such systems, the explicit use of human knowledge and causal relationships may be useful. In general, humans are fast in understanding regime changes, for example, the changes caused by the COVID-19 pandemic. In any case, a full understanding may remain hard to achieve by machine learning, as illustrated by the Chinese room argument [62].

The explainability of machine learning and artificial intelligence is another issue. Machine learning can be distinguished into various types, such as supervised learning, unsupervised learning and reinforcement learning. The algorithms used may be shallow or deep, with many free parameters. Technically, they can all be regarded as a way to automatically specify a hidden variable model Λ that gives good predictions based on well-defined input data [60]. With deep learning approaches, the parameter space that makes up Λ can be very large. Although the numerical values may be set automatically to provide good forecasts on training and test data, the interpretability and explainability of such hidden variable models can be poor. Context-adaptive procedures, i.e., systems that construct contextual explanatory models for classes of real-world phenomena, would be desirable [63,64,65]. Linking probabilistic learning methods with representations of known causal relationships can improve on methods restricted to data mining, which are based only on correlations. Plausible causal models or a reasonably conjectured hypothesis in the theory of probably-approximately-correct learning may, for example, serve as a starting point to restrict the space of possible models [41,66,67,68,69,70]. In the same way as hyperparameters in machine learning can be set manually or determined by means of a routine, a human specialist could set up simple plausible causal relationships, for example, using directed acyclical graphs for Baysian networks [71,72,73,74], and Bell inequalities could be used to test them automatically, as the Baysian networks would place restrictions on the joint distribution of the observed variables [75,76]. Causal models that are not ruled out by the available data would be available for statistical parameter fitting by existing machine learning algorithms and could provide explainable causal connections.

Without human input, machine learning is limited. As shown in [77], standard causal discovery algorithms that try to infer causal relationships from correlations cannot distinguish correlations that satisfy Bell inequalities from those correlations that violate those inequalities.

### 6.4. Connection Strength in Quantum Systems, Free Choice and Locality

Modern discussions of Bell’s work in quantum foundations focus less on hidden variable models and instead summarize Bell’s theorem by saying that the combination of three assumptions, namely, *locality*, *free choice* and *realism*, force $|S_{i}|⩽2$. As we can observe violations of these inequalities experimentally, at least one of the three assumptions has to be given up. Unsurprisingly, this is a controversial and exciting statement, because these three terms hold cherished meanings in everyday language and intuitive theories about nature, albeit not always precisely defined ones.

Several ways of dealing with the conundrum are possible. At the most basic level, one may decide not to care too much, because the formalism of quantum theory already allows predictions for measurable outcomes correctly and reconciles conservation laws with a probabilistic description of physical reality in a mathematically elegant way. If one seeks a more complete explanation, one may say that nature tells us that the notion that a system of particles has pre-existing values for all possible measurements has to be rejected. Under this view the measurement context becomes important, as the results will depend on how a measurement is applied. It is not always possible to specify a single probability space in advance for all possible measurements; rather, “quantum probabilities” appear based on classical probability according to the conditions of different possible experimental settings [22]. Interpreting the meaning of a quantum state, and the question of whether it expresses the objective underlying physical state of a system or rather the possible measurement outcomes and information, is still a matter of debate [19,21,23]. Contextuality becomes an important concept [25,28,78,79,80,81,82,83] and it can be formalized to some degree, for example, by requiring predictive completeness, which means that the quantum state has to be completed by linking it with a measurement operator as well [29].

Alternatively, Bell’s approach may be considered as pointing to something new, which is responsible for the observed connection strength. One may use it as a starting point to reconsider the role of time and retrocausality [84,85], restrictions of free choice [86,87,88], superdeterminism [89,90], collapse theories [91,92,93] or to speculate about what quantum entanglement tells us about the fabric of spacetime [94,95,96].

In any case, one needs to keep in mind that in Bell’s approach, the terms free choice and locality are essentially used as names for certain equations on the basis of the hidden variable space Λ and a joint probability measure: Equation (11) is named *(i) locality* and Equation (12) is referred to as *(ii) free choice* or even *free will*. In response to [97], just as Valerio Scarani has used the title *Bell nonlocality* for his book [24], the longer term *Bell free choice* may be more fitting for (ii). Essentially, (ii) is a statistical independence condition. As shown in [27], assumptions (i) and (ii) can, on the basis of *(iii) realism*, be proven to be equivalent, when it comes to the optimal strategies to simulate a desired joint statistic P(a,b|xy). This is surprising as (i) and (ii) would appear to be unconnected concepts in everyday language. If one imagines a universe with an infinite speed of light, the locality principle would disappear, but there is no reason why this should affect the question of free will. Thus, in a sense, the mathematical equivalence of violations of (i) and (ii) can also be taken as an argument for making a clearer distinction between the terms free choice and locality in everyday language, in order to differentiate from their use in the sense of Bell.

## Figures and Tables

**Table 1:** The following table illustrates a specific strategy, using Cheat 1 and communication in each round.

*x*	Message	Regime	*a*	*b*	ab
0	None	xy∈{00,01}	+1	+1	+1
1	Yes	xy=10	+1	+1	+1
1	Yes	xy=11	+1	−1	−1

**Table 2:** The following table illustrates a specific strategy using Cheat 2, where both Alice and Bob know the regime in each round.

*x*	*y*	Regime Known	*a*	*b*	ab
0	0	yes	+1	+1	+1
0	1	yes	+1	+1	+1
1	0	yes	+1	+1	+1
1	1	yes	+1	−1	−1

**Table 3:** The following table shows the different situations for a specific strategy using Cheat 1, where Alice sends text messages in some rounds in which she has received the question x=1.

*x*	Message	Regime	*a*	*b*	ab	Round In
0	None	xy∈{00,01,10,11}	+1	+1	+1	$\bar{T}$
1	Yes	xy=10	+1	+1	+1	*T*
1	Yes	xy=11	+1	−1	−1	*T*

**Table 4:** The following table illustrates a specific strategy using Cheat 2, where Alice and Bob know the regime in some rounds.

*x*	*y*	Regime Known	*a*	*b*	ab	Round In
0	0	irrelevant	+1	+1	+1	$K\cup \bar{K}$
0	1	irrelevant	+1	+1	+1	$K\cup \bar{K}$
1	0	irrelevant	+1	+1	+1	$K\cup \bar{K}$
1	1	no	+1	+1	+1	$\bar{K}$
1	1	yes	+1	−1	−1	*K*

**Table 5:** The following table illustrates the behavior of Alice and Bob. They both conduct only typical activities, except Bob behaves atypically on days when he receives confirmation from someone with private information confirming that market rumors are true.

*x*	*y*	Situation	*a*	*b*	ab
0	0	no large order, no rumors	+1	+1	+1
0	1	no large order, but (unfounded) rumors	+1	+1	+1
1	0	large order, but no rumors	+1	+1	+1
1	1	large order and rumors, but no confirmation to Bob	+1	+1	+1
1	1	large order and rumors and confirmation to Bob (“K”)	+1	−1	−1

**Table 6:** The following table illustrates the behavior in the case that Bob sometimes reacts to rumors by executing unusual transactions in the market and never receives information from the private side.

*x*	*y*	Situation	*a*	*b*	ab	Prob.
0	0	no large order, no rumors	+1	+1	+1	100%
0	1	no large order, but (unfounded) rumors	+1	−1	−1	*p*
0	1	no large order, but (unfounded) rumors	+1	+1	+1	1−p
1	0	large order, but no rumors	+1	+1	+1	100%
1	1	large order, (true) rumors	+1	−1	−1	$p^{\prime }$
1	1	large order, (true) rumors	+1	+1	+1	$1-p^{\prime }$

## Data Availability

Not applicable.

## References

[1] Bell J.S. (1964). On the Einstein-Podolsky-Rosen paradox. Physics.

[2] Bell J.S. (1966). On the Problem of Hidden Variables in Quantum Mechanics. Rev. Mod. Phys..

[3] Bell J.S. (1970). Introduction to the Hidden Variables Question.

[4] Bell J., Shimony A., Horne M., Clauser J. (1985). An Exchange on Local Beables. Dialectica.

[5] Bell J.S. (1987). Free variables and local causality. Speakable and Unspeakable in Quantum Mechanics.

[6] Bell J.S. (1987). Speakable and Unspeakable in Quantum Mechanics.

[7] Aspect A., Grangier P., Roger G. (1981). Experimental Tests of Realistic Local Theories via Bell’s Theorem. Phys. Rev. Lett..

[8] Aspect A., Dalibard J., Roger G. (1982). Experimental Test of Bell’s Inequalities Using Time-Varying Analyzers. Phys. Rev. Lett..

[9] Weihs G., Jennewein T., Simon C., Weinfurter H., Zeilinger A. (1998). Violation of Bell’s Inequality under Strict Einstein Locality Conditions. Phys. Rev. Lett..

[10] Shalm L.K., Meyer-Scott E., Christensen B.G., Bierhorst P., Wayne M.A., Stevens M.J., Gerrits T., Glancy S., Hamel D.R., Allman M.S. (2015). Strong Loophole-Free Test of Local Realism. Phys. Rev. Lett..

[11] Hensen B., Bernien H., Dreau A.E., Reiserer A., Kalb N., Blok M.S., Ruitenberg J., Vermeulen R.F.L., Schouten R.N., Abellan C. (2015). Loophole-free Bell inequality violation using electron spins separated by 1.3 kilometres. Nature.

[12] Giustina M., Versteegh M.A.M., Wengerowsky S., Handsteiner J., Hochrainer A., Phelan K., Steinlechner F., Kofler J., Larsson J.A., Abellán C. (2015). Significant-Loophole-Free Test of Bell’s Theorem with Entangled Photons. Phys. Rev. Lett..

[13] Abellán C., Acín A., Alarcón A., Alibart O., Andersen C.K., Andreoli F., Beckert A., Beduini F.A., Bendersky A., Bentivegna M. (2018). Challenging local realism with human choices. Nature.

[14] Suppes P., Zanotti M. (1981). When are probabilistic explanations possible?. Synthese.

[15] Leggett A.J., Garg A. (1985). Quantum mechanics versus macroscopic realism: Is the flux there when nobody looks?. Phys. Rev. Lett..

[16] Selleri F. (1990). Quantum Paradoxes and Physical Reality.

[17] Leggett A.J. (2003). Nonlocal Hidden-Variable Theories and Quantum Mechanics: An Incompatibility Theorem. Found. Phys..

[18] Klyachko A.A., Can M.A., Binicioğlu S., Shumovsky A.S. (2008). Simple Test for Hidden Variables in Spin-1 Systems. Phys. Rev. Lett..

[19] Pusey M.F., Barrett J., Rudolph T. (2012). On the reality of the quantum state. Nat. Phys..

[20] Maudlin T. (2014). What Bell did. J. Phys. A Math. Theor..

[21] Aspect A. (2015). Closing the Door on Einstein and Bohr’s Quantum Debate. Physics.

[22] Khrennikov A. (2015). CHSH Inequality: Quantum Probabilities as Classical Conditional Probabilities. Found. Phys..

[23] Cabello A., Lombardi O., Fortin S., Holik F., López C. (2017). Interpretations of Quantum Theory: A Map of Madness. What Is Quantum Information?.

[24] Scarani V. (2019). Bell Nonlocality.

[25] Grangier P. (2021). Contextual Inferences, Nonlocality, and the Incompleteness of Quantum Mechanics. Entropy.

[26] Khrennikov A. (2021). Is the Devil in h?. Entropy.

[27] Blasiak P., Pothos E.M., Yearsley J.M., Gallus C., Borsuk E. (2021). Violations of locality and free choice are equivalent resources in Bell experiments. Proc. Natl. Acad. Sci. USA.

[28] Kupczynski M. (2021). Contextuality-by-Default Description of Bell Tests: Contextuality as the Rule and Not as an Exception. Entropy.

[29] Grangier P. (2021). Completing the Quantum Formalism in a Contextually Objective Framework. Found. Phys..

[30] Contreras-Tejada P., Scarpa G., Kubicki A., Brandenburger A., Mura P. (2021). Observers of quantum systems cannot agree to disagree. Nat. Commun..

[31] Gisin N. (2014). Quantum Chance: Nonlocality, Teleportation and Other Quantum Marvels.

[32] López-Incera A., Hartmann A., Dür W. (2020). Encrypt me! A game-based approach to Bell inequalities and quantum cryptography. Eur. J. Phys..

[33] Yuen H. The Complexity of Entanglement; Lecture Notes for CSC2429/MAT1751 (Fall 2020) at the University of Toronto. http://www.henryyuen.net/fall2020/complexity_of_entanglement_notes.pdf.

[34] Welsch B., Thron C. (2021). The ’Quantum Game Show’: A Very Simple Explanation of Bell’s Theorem in Quantum Mechanics. https://ssrn.com/abstract=3956512.

[35] Summers S.J., Werner R. (1987). Maximal violation of Bell’s inequalities is generic in quantum field theory. Commun. Math. Phys..

[36] McKague M., Yang T.H., Scarani V. (2012). Robust self-testing of the singlet. J. Phys. A Math. Theor..

[37] Grilo A. (2020). A simple protocol for verifiable delegation of quantum computation in one round. arXiv.

[38] Clauser J.F., Horne M.A., Shimony A., Holt R.A. (1969). Proposed Experiment to Test Local Hidden-Variable Theories. Phys. Rev. Lett..

[39] Gill R.D. (2014). Statistics, Causality and Bell’s Theorem. Stat. Sci..

[40] Brunner N., Cavalcanti D., Pironio S., Scarani V., Wehner S. (2014). Bell nonlocality. Rev. Mod. Phys..

[41] Pearl J. (2009). Causality: Models, Reasoning, and Inference.

[42] Popescu S., Rohrlich D. (1994). Quantum Nonlocality as an Axiom. Found. Phys..

[43] Aspect A., Bertlmann R.A., Zeilinger A. (2002). Bell’s Theorem: The Naive View of an Experimentalist. Quantum [Un]speakables: From Bell to Quantum Information.

[44] Toner B.F., Bacon D. (2003). Communication Cost of Simulating Bell Correlations. Phys. Rev. Lett..

[45] Waddup O., Blasiak P., Yearsley J.M., Wojciechowski B.W., Pothos E.M. (2021). Sensitivity to Context in Human Interactions. Mathematics.

[46] Pearle P.M. (1970). Hidden-Variable Example Based upon Data Rejection. Phys. Rev. D.

[47] Larsson J.A. (2014). Loopholes in Bell inequality tests of local realism. J. Phys. A Math. Gen..

[48] Adenier G., Khrennikov A. (2017). Test of the no-signaling principle in the Hensen loophole-free CHSH experiment. Fortschr. Phys..

[49] Hensen B., Kalb N., Blok M.S., Dréau A.E., Reiserer A., Vermeulen R.F.L., Schouten R.N., Markham M., Twitchen D.J., Goodenough K. (2016). Loophole-free Bell test using electron spins in diamond: Second experiment and additional analysis. Sci. Rep..

[50] Liang Y.C., Zhang Y. (2019). Bounding the Plausibility of Physical Theories in a Device-Independent Setting via Hypothesis Testing. Entropy.

[51] Robins J.M., VanderWeele T.J., Gill R.D. (2015). A proof of Bell’s inequality in quantum mechanics using causal interactions. Scand. J. Stat. Theory Appl..

[52] Pearl J. On the testability of causal models with latent and instrumental variables. Proceedings of the Eleventh Conference on Uncertainty in Artificial Intelligence.

[53] Bonet B. (2001). Instrumentality Tests Revisited. Proceedings of the Seventeenth Conference on Uncertainty in Artificial Intelligence.

[54] Einstein A., Podolsky B., Rosen N. (1935). Can Quantum-Mechanical Description of Physical Reality Be Considered Complete?. Phys. Rev..

[55] Hastie T., Tibshirani R., Friedman J. (2009). The Elements of Statistical Learning: Data Mining, Inference and Prediction.

[56] Jordan M.I., Mitchell T.M. (2015). Machine learning: Trends, perspectives, and prospects. Science.

[57] Jayanth Balaji A., Harish Ram D., Nair B.B. (2018). Applicability of Deep Learning Models for Stock Price Forecasting: An Empirical Study on BANKEX Data. Procedia Comput. Sci..

[58] Henrique B.M., Sobreiro V.A., Kimura H. (2019). Literature review: Machine learning techniques applied to financial market prediction. Expert Syst. Appl..

[59] Mashrur A., Luo W., Zaidi N.A., Robles-Kelly A. (2020). Machine Learning for Financial Risk Management: A Survey. IEEE Access.

[60] Janiesch C., Zschech P., Heinrich K. (2021). Machine learning and deep learning. Electron. Mark..

[61] Singh A.K., Gupta P., Thakur N. (2021). An Empirical Research and Comprehensive Analysis of Stock Market Prediction using Machine Learning and Deep Learning techniques. IOP Conf. Ser. Mater. Sci. Eng..

[62] Searle J.R. (1980). Minds, brains, and programs. Behav. Brain Sci..

[63] Gilpin L.H., Bau D., Yuan B.Z., Bajwa A., Specter M.A., Kagal L. Explaining Explanations: An Overview of Interpretability of Machine Learning. Proceedings of the 2018 IEEE 5th International Conference on Data Science and Advanced Analytics (DSAA).

[64] Phillips P., Hahn A., Fontana P., Broniatowski D., Przybocki M. (2020). Four Principles of Explainable Artificial Intelligence (Draft).

[65] Holzinger A. From Machine Learning to Explainable AI. Proceedings of the 2018 World Symposium on Digital Intelligence for Systems and Machines (DISA).

[66] Pearl J. (1988). Probabilistic Reasoning in Intelligent Systems.

[67] Valiant L.G. (1984). A Theory of the Learnable. Commun. ACM.

[68] Valiant L.G. (2013). Probably Approximately Correct: Nature’s Algorithms for Learning and Prospering in a Complex World.

[69] Kearns M., Li M. (1993). Learning in the Presence of Malicious Errors. SIAM J. Comput..

[70] Kearns M., Vazirani U. (1994). An Introduction to Computational Learning Theory.

[71] Heckerman D., Geiger D., Chickering D.M. (1995). Learning Bayesian networks: The combination of knowledge and statistical data. Mach. Learn..

[72] Spirtes P., Glymour C., Scheines R. (2000). Causation, Prediction, and Search.

[73] Jensen F.V., Nielsen T.D. (2007). Bayesian Networks and Decision Graphs.

[74] Evans R.J. (2016). Graphs for Margins of Bayesian Networks. Scand. J. Statist..

[75] Tian J., Pearl J., Darwiche A., Friedman N. (2002). On the Testable Implications of Causal Models with Hidden Variables. Uncertainty in Artificial Intelligence, Proceedings of the Eighteenth Conference, Alberta, Canada, 1–4 August 2002.

[76] Steeg G.V., Galstyan A. (2011). A Sequence of Relaxations Constraining Hidden Variable Models. Proceedings of the Twenty-Seventh Conference on Uncertainty in Artificial Intelligence.

[77] Wood C.J., Spekkens R.W. (2015). The lesson of causal discovery algorithms for quantum correlations: Causal explanations of Bell-inequality violations require fine-tuning. New J. Phys..

[78] Spekkens R.W. (2005). Contextuality for preparations, transformations, and unsharp measurements. Phys. Rev. A.

[79] Khrennikov A. (2009). Contextual Approach to Quantum Formalism.

[80] Cabello A. (2010). Proposed test of macroscopic quantum contextuality. Phys. Rev. A.

[81] Dzhafarov E.N., Kujala J.V. (2016). Context–content systems of random variables: The Contextuality-by-Default theory. J. Math. Psychol..

[82] Abramsky S., Barbosa R.S., Mansfield S. (2017). Contextual Fraction as a Measure of Contextuality. Phys. Rev. Lett..

[83] Budroni C., Cabello A., Gühne O., Kleinmann M., Larsson J.Å. (2021). Quantum Contextuality. arXiv.

[84] Aharonov Y., Vaidman L., Muga J.G., Sala Mayato R., Egusquiza I.L. (2002). The Two-State Vector Formalism of Quantum Mechanics. Time in Quantum Mechanics.

[85] Leifer M.S., Pusey M.F. (2017). Is a time symmetric interpretation of quantum theory possible without retrocausality?. Proc. R. Soc. A.

[86] Brans C.H. (1988). Bell’s theorem does not eliminate fully causal hidden variables. Int. J. Theor. Phys..

[87] Hall M.J.W. (2011). Relaxed Bell inequalities and Kochen-Specker theorems. Phys. Rev. A.

[88] Hall M.J.W., Branciard C. (2020). Measurement-dependence cost for Bell nonlocality: Causal versus retrocausal models. Phys. Rev. A.

[89] ’t Hooft G. (2016). The Cellular Automaton Interpretation of Quantum Mechanics.

[90] Hossenfelder S., Palmer T. (2020). Rethinking Superdeterminism. Front. Phys..

[91] Tumulka R. (2006). A Relativistic Version of the Ghirardi–Rimini–Weber Model. J. Stat. Phys..

[92] Gisin N. (2011). Impossibility of covariant deterministic nonlocal hidden-variable extensions of quantum theory. Phys. Rev. A.

[93] Esfeld M., Gisin N. (2014). The GRW Flash Theory: A Relativistic Quantum Ontology of Matter in Space-Time?. Philos. Sci..

[94] Maldacena J., Susskind L. (2013). Cool horizons for entangled black holes. Fortsch. Phys..

[95] Susskind L. (2016). ER = EPR, GHZ, and the consistency of quantum measurements. Fortsch. Phys..

[96] Dai D.C., Minic D., Stojkovic D., Fu C. (2020). Testing the ER = EPR conjecture. Phys. Rev. D.

[97] Kupczynski M. (2021). A comment on: The violations of locality and free choice are equivalent resources in Bell experiments. arXiv.

